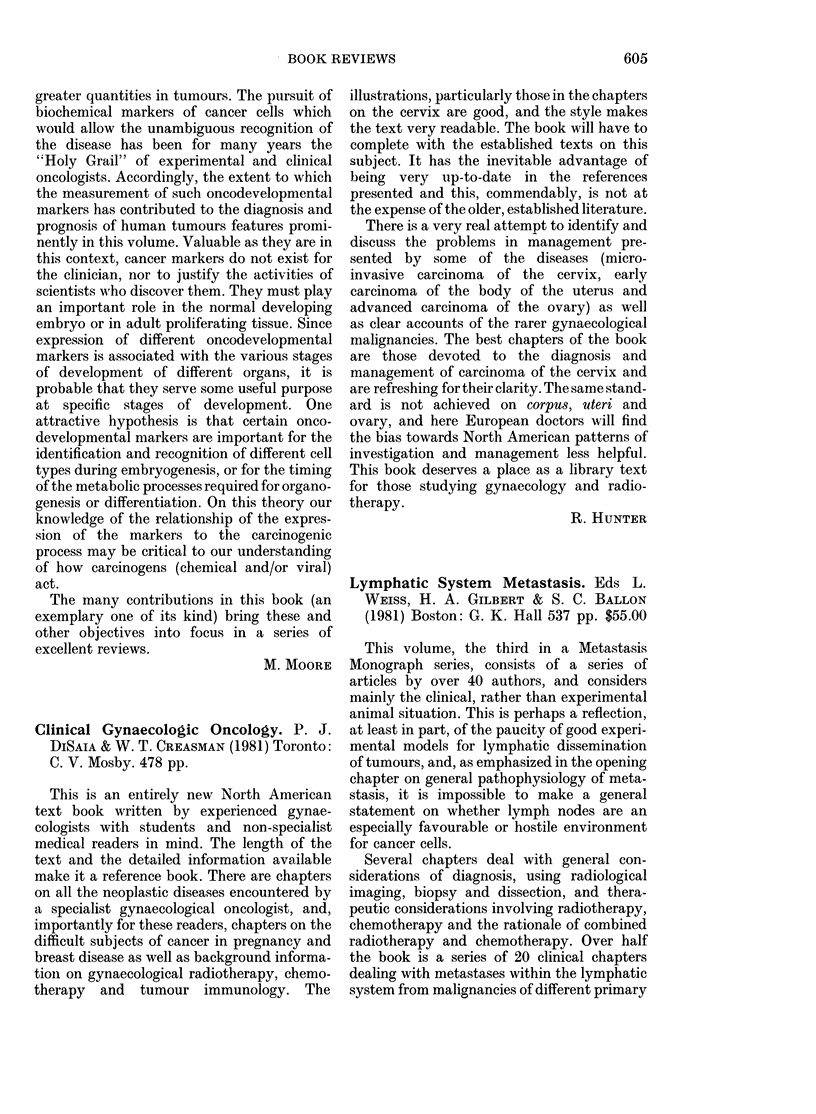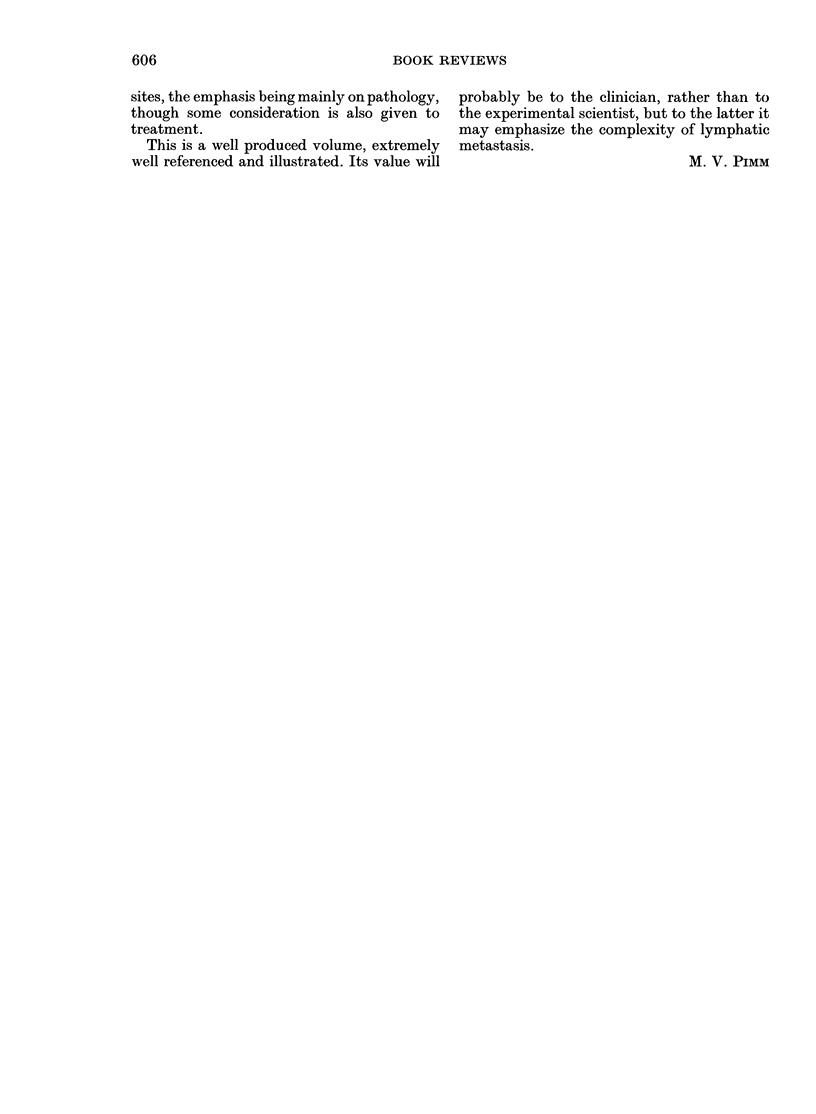# Lymphatic System Metastasis

**Published:** 1981-10

**Authors:** M. V. Pimm


**Lymphatic System Metastasis.** Eds L. WEISS, H. A. GILBERT & S. C. BALLON (1981) Boston: G. K. Hall 537 pp. $55.00

This volume, the third in a Metastasis Monograph series, consists of a series of articles by over 40 authors, and considers mainly the clinical, rather than experimental animal situation. This is perhaps a reflection, at least in part, of the paucity of good experimental models for lymphatic dissemination of tumours, and, as emphasized in the opening chapter on general pathophysiology of metastasis, it is impossible to make a general statement on whether lymph nodes are an especially favourable or hostile environment for cancer cells.

Several chapters deal with general considerations of diagnosis, using radiological imaging, biopsy and dissection, and therapeutic considerations involving radiotherapy, chemotherapy and the rationale of combined radiotherapy and chemotherapy. Over half the book is a series of 20 clinical chapters dealing with metastases within the lymphatic system from malignancies of different primary sites, the emphasis being mainly on pathology, though some consideration is also given to treatment.

This is a well produced volume, extremely well referenced and illustrated. Its value will probably be to the clinician, rather than to the experimental scientist, but to the latter it may emphasize the complexity of lymphatic metastasis.